# Metastatic pulmonary adenocarcinoma after 20 years of primary pancreatic adenocarcinoma resection: A case report and literature review

**DOI:** 10.1016/j.amsu.2022.104150

**Published:** 2022-07-13

**Authors:** Osama N. Dukmak, Almotazbellah Zeer, Fajr M.A. Sarhan, Mohammad Eid Al mohtasib, Yousef Abu Asbeh

**Affiliations:** a Faculty of Medicine, Al-Quds University, Jerusalem, Palestine; b Thoracic Department, Ahli Hospital, Hebron, Palestine

**Keywords:** Pancreatic adenocarcinoma, Pulmonary metastasis, Late recurrence, Case report, CA19-9

## Abstract

**Introduction:**

Recurrency of Pancreatic adenocarcinoma after resection can be as high as 85%. Most of the recurrences happen within two years of pancreatic resection and may be local or present as a metastatic disease.

**Clinical presentation:**

Herein, we report a patient who presented with metastatic pulmonary adenocarcinoma after 20 years of curative resection of pancreatic adenocarcinoma. Histopathology of the pulmonary mass confirms the diagnosis as a metastatic adenocarcinoma of gastrointestinal origin.

**Conclusion:**

Despite the length of the disease-free period, lung metastasis of pancreatic cancer is the most likely diagnosis according to the clinical course, histopathology and biochemical tumor marker. Tumor marker Ca19-9 is very sensitive and reliable test to detect recurrency of pancreatic cancer even if the imaging modalities cannot detect the tumor.

## Highlights

• Recurrency of Pancreatic adenocarcinoma after resection can be as high as 85%.
• Most of the recurrences happen within two years of pancreatic resection but recurrence may occur even after 20 years.
• Tumor marker Ca19-9 is very sensitive and reliable test to detect recurrency of pancreatic cancer.
• In patients with an isolated pulmonary metastasis overall survival range between 23 months and 92.3 month.

## 1 Introduction

Pancreatic cancer is one of the leading causes of cancer mortality and one of the most lethal malignant neoplasms worldwide [1,2].

Surgery is the curative option for the early stage of pancreatic cancer; nonetheless, most patients experience a recurrence after surgery and die as a consequence of it [3].

In Patients who have a respectable disease and undergo curative surgery, recurrences occur in the early years after surgery. These recurrences may be isolated to the pancreatic bed or present as a distant metastatic disease. In general, recurrence decreases the overall survival rate and worsens the prognosis [4].

We describe, herein, a case of late a lung metastasis, 20 years after resection for pancreatic adenocarcinoma by Whipple procedure without evidence of recurrent local pancreatic disease.

## 2 Case presentation

A 73-year-old smoker male patient who initially underwent successful resection of pancreatic adenocarcinoma 20 years ago, presented with dyspnea and cough that progressively worsened in the past months.

The patient's symptoms started around eight months ago, when he developed a chronic cough which was dry then became productive. One month ago, since symptoms did not improve, he sought medical attention in a local hospital.

Medical history includes diabetes mellitus controlled by insulin. Also, he had undergone Whipple procedure for his pancreatic adenocarcinoma 20 years ago, in which half of the stomach, duodenum, upper jejunum, head of pancreas, uncinate process of the pancreas and common bile duct (CBD) were resected. As patient reported, he did not receive any adjuvant or neoadjuvant chemotherapy. Unfortunately, the histopathology for the procedure is not available due to poor documentation 20 years ago. We only had a report stating the result.

**Fig. 1:**
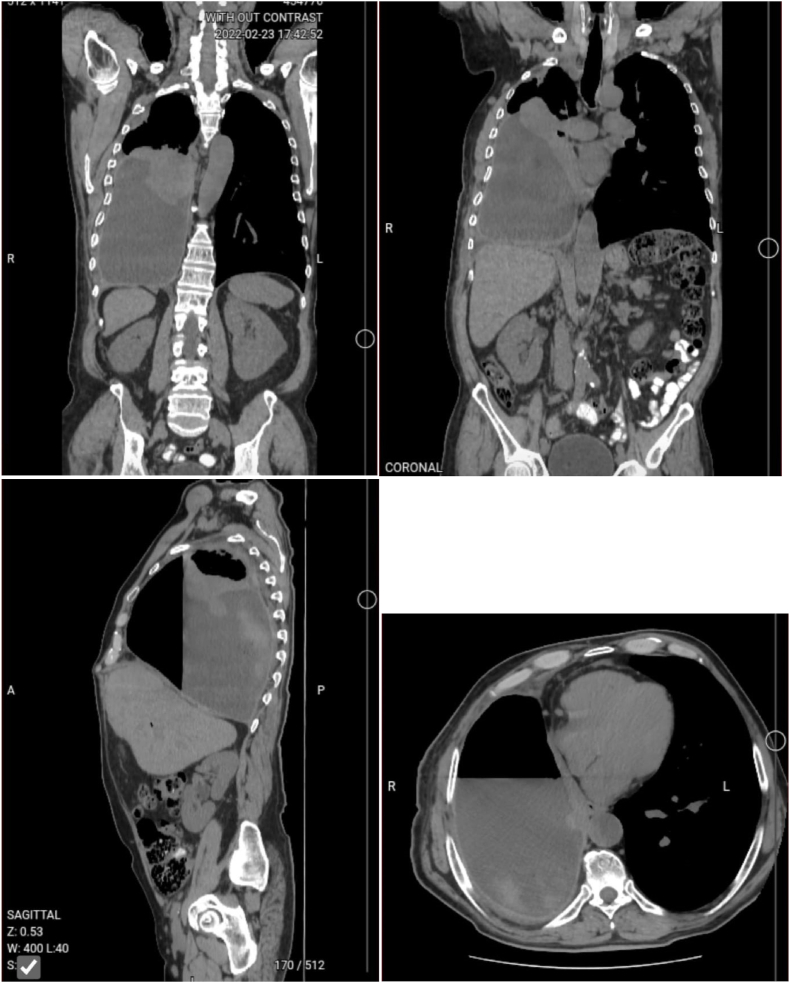
CT scan of the chest showing: Large right side pleural effusion causing compression atelectasis and loss of volume of right lung.

Chest x ray and CT scan (Fig. 1) revealed multiloculated right sided pleural effusion with a trapped lung. Chest tube was applied and failed to drain the effusion. Thus, he was referred to the Thoracic Surgery ward for further evaluation and treatment.

The patient underwent Video-assisted thoracoscopic surgery (VATS) on the right thorax. All pleural fluids drained, there was a high suspicion for malignant effusion. Cytology for pleural effusion was unremarkable. The patient then underwent complete investigation and work up, including upper and lower gastrointestinal endoscopy, whole body CT scan with and without contrast and Positron emission tomography (PET) scan, all of these studies did not detect any abnormality.

The tumor marker CA19-9 was elevated more than 1200 U/ml (reference range: 0–37 U/ml). The CEA, routine blood count and other biochemical tests were all within the normal limits.

Due to very high elevations in CA19-9, the patient was then referred for deep pleural biopsy.

**Fig. 2:**
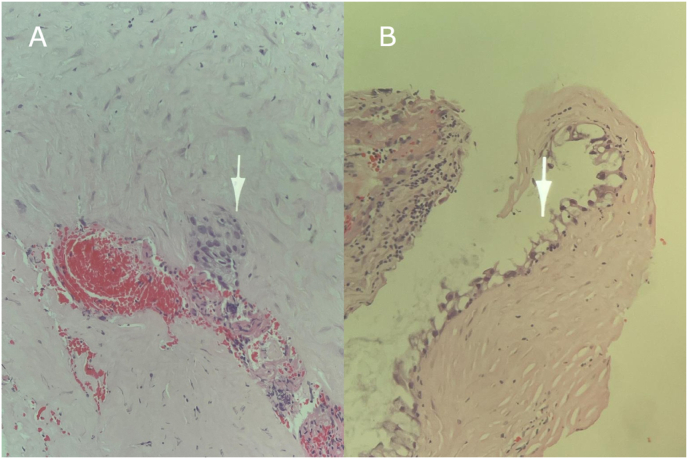
Histopathology from pleural biopsy, Hematoxylin–eosin staining, 20× magnification, serially sectioned, (B is deeper level than A), Histology reveals features of chronic inflammation along with a distinct focus showing atypical glandular structures with mucin secreting glands.

Pleural biopsy taken was serially sectioned and histological examination (Fig. 2) revealed pleural tissue with a marked chronic inflammation along with distinct focus showing mucin secreting glands highlighted by CK7 and CK20 immunostaining. CDX2 was negative, these findings were consistent with metastatic adenocarcinoma of a gastrointestinal origin.

Despite the length of time, lung metastasis of pancreatic cancer was the most likely diagnosis according to the clinical course and biochemical tumor marker.

## 3 Discussion

Despite surgical resection and adjuvant therapy, pancreatic adenocarcinoma remains one of the most incurable cancers which has a poor overall survival and significant recurrence rates [5].

The extra-pancreatic recurrence (including the liver, peritoneum, lung and retroperitoneum) is the most common after resection of the pancreatic cancer. On the other hand, locoregional recurrence at the resection site comes in the second order of recurrences rates. Generally, most recurrence occur in the early post operative period [6].

Although metachronous pulmonary metastases following pancreatic resection are the most common extra abdominal location of the pancreatic adenocarcinoma recurrence, this form of recurrent disease is very uncommon [7].

The disease-free survival (DFS), and overall survival (OS) rates are the best tools to study the recurrence after the pancreatic cancer resection. In patients with an isolated pulmonary metastasis median DFS ranges between 10.5 months and 52.4 months while the OS (overall survival) ranges 23 months and 92.3 months [7].

In the literature, there is a reported case with long DFS following distal pancreatectomy, About 156 months (13 years) [8]. Those patient with long disease-free interval and solitary gradual course of the disease recurrence are more likely to benefit from surgical resection [9].

We report a patient who successfully underwent Whipple pancreatectomy for pancreatic adenocarcinoma 20 years ago. Recently, he was diagnosed with metastatic pulmonary adenocarcinoma. Table 1 presents a review of patients with metachronous lung metastasis from pancreatic adenocarcinoma after curative resection.

**Table 1:** Review of patient with metachronous lung metastasis from Pancreatic Adenocarcinoma after Curative resection.

#	Author	Number of patients	Age in years	Sex	Adjuvant Chemotherapy after pancreatic resection	Disease free survival (DFS)	Follow up after Metachronous pulmonary metastasis a
1	Kitasato et al. [8]	1	46	F	Cisplatin + 5-fluorouracil	13 years	Alive, 14 months
2	Miyasaka et al. [12]	1	70	F	gemcitabine	16 months	Alive, 5 years
3	Falkernstern-Ge et al. [13]	1	73	F	gemcitabine	5 years	Alive, 3 years
4	Uesato et al. [14]	1	75	M	gemcitabine	4 years/7 years *^b^	Alive, 12 years
6	Nakajima et al. [15]	17	75 (52–81)	6 M9 F	Gemcitabine (10 patients)	3.6 years (0.6–8)	37 (9–93) months
5	Our case	1	73	M	Did not receive	20 years	Alive

a Time After diagnosis of pulmonary metastasis and date of the death or last follow-up, whichever occurred first.

b There were two recurrences after pancreatic surgery, after 4 years and 11 years of primary pancreatic resection.

Comprehensive workup and investigations including upper and lower endoscopy, whole body CT scan and PET scan were done and were normal. Nevertheless, tumor markers showed significantly elevated CA19-9 (more than 1200 U/ml) which was consistent with metastatic pancreatic cancer.

CA19-9 is considered as reliable biomarker for detecting the recurrence of pancreatic adenocarcinoma [10].

Video-assisted thoracic surgery (VATS) is safer than conventional thoracotomy, also, it provides better functional recovery especially in patients with high morbidity and mortality. VATS is currently accepted as an appropriate procedure for selected patients with early-stage non-small-cell lung cancer (NSCLC) [11].

Although the time interval between the primary pancreatic cancer and lung cancer is extremely long, we have to keep in mind the probability for the lung cancer to be metastatic due to the pathology report supporting a gastrointestinal origin of the tumor.

## 4 Conclusion

Despite the length of the disease-free period, lung metastasis of pancreatic cancer is the most likely diagnosis according to the clinical course, histopathology and biochemical tumor marker.

Tumor marker Ca19-9 is very sensitive and reliable test to detect recurrency of pancreatic cancer even if the imaging modalities cannot detect the tumor.

## Declaration of competing interest

There is no conflict of interest to declare.

## Provenance and peer review

Not commissioned, externally peer-reviewed.

## Methods

This case has been reported in line with SCARE Criteria [16].

## Ethical approval

The study is exempt from ethical approval in our institution.

## Sources of funding

No funding or grant support.

## Author contribution

Data collection: Yousef Abu Asbeh, Mohammad Eid Al mohtasib, Writing the manuscript: Osama Dukmak, Almotazbellah Zeer, Study concept or design: Osama Dukmak, Almotazbellah Zeer, Yousef abu asbeh, Mohammad eid al mohtasib, Review & editing the manuscript: Osama Dukmak, Yousef abu asbeh.

## Registration of research studies

Not applicable.

## Guarantor

Dr. Yousef abu asbeh.

## Consent

Written informed consent was obtained from the patient for publication of this case report and accompanying images. A copy of the written consent is available for review by the Editor-in-Chief of this journal on request.
